# Current Pharmaceutical Strategies in the Management of Persistent Pulmonary Hypertension of the Newborn (PPHN): A Comprehensive Review of Therapeutic Agents

**DOI:** 10.7759/cureus.70307

**Published:** 2024-09-27

**Authors:** Divyanshi Kaplish, Jayant D Vagha, Sachin Rathod, Aditya Jain

**Affiliations:** 1 Pediatrics, Jawaharlal Nehru Medical College, Datta Meghe Institute of Higher Education and Research, Wardha, IND; 2 Obstetrics and Gynecology, Jawaharlal Nehru Medical College, Datta Meghe Institute of Higher Education and Research, Wardha, IND

**Keywords:** extracorporeal membrane oxygenation, inhaled nitric oxide, neonatal care, newborn, persistent pulmonary hypertension, pharmacotherapy

## Abstract

Persistent Pulmonary Hypertension of the Newborn (PPHN) is a life-threatening condition characterized by the failure of normal circulatory transition after birth, leading to sustained pulmonary hypertension and severe hypoxemia. Despite advancements in neonatal care, PPHN remains a significant cause of morbidity and mortality among newborns, particularly in full-term and near-term infants. This review provides a comprehensive overview of current pharmaceutical strategies for managing PPHN, focusing on various therapeutic agents' mechanisms, efficacy, and safety. Key interventions include inhaled nitric oxide, which has become the standard treatment for reducing pulmonary vascular resistance, alongside prostacyclin analogs, phosphodiesterase inhibitors, and endothelin receptor antagonists. Additionally, extracorporeal membrane oxygenation (ECMO) is highlighted as a critical intervention for severe, refractory cases. The review also discusses emerging therapies and the potential role of personalized medicine in improving treatment outcomes. Despite the progress made, challenges remain, including the timely diagnosis of PPHN and the need for accessible treatments in resource-limited settings. As research continues to uncover the underlying pathophysiology of PPHN, it is crucial to develop more targeted and effective pharmaceutical strategies. This review aims to inform clinicians and researchers of the current state of PPHN management and the ongoing advancements that may shape future therapeutic approaches.

## Introduction and background

Persistent Pulmonary Hypertension of the Newborn (PPHN) is a critical condition that arises when the normal circulatory transition after birth fails, leading to sustained high pulmonary vascular resistance [1]. In a healthy newborn, the pulmonary arteries rapidly dilate after birth, allowing blood to flow into the lungs for oxygenation. However, in infants with PPHN, this process is disrupted, causing blood to bypass the lungs through fetal circulatory pathways like the ductus arteriosus and foramen ovale [1]. This results in severe hypoxemia and respiratory distress, posing significant risks to the infant’s health. The underlying mechanisms of PPHN are complex, involving abnormal pulmonary vascular remodeling, endothelial dysfunction, and altered vasoreactivity, which together contribute to persistently elevated pulmonary artery pressure [2].

The incidence of PPHN is approximately one to two per 1,000 live births, with variations depending on population and diagnostic criteria. It predominantly affects full-term or near-term infants and is more common in males [3]. Despite advances in neonatal care, PPHN remains a significant cause of neonatal morbidity and mortality, particularly in settings with limited access to advanced therapeutic interventions [2]. Several factors increase the risk of developing PPHN, including maternal conditions like diabetes and obesity, perinatal complications such as meconium aspiration syndrome and congenital diaphragmatic hernia, and neonatal conditions like sepsis. These risk factors, along with any condition that induces hypoxia or acidosis, can precipitate the onset of PPHN in newborns [2].

Early diagnosis and management of PPHN are crucial in preventing severe complications and improving outcomes. The condition is associated with a high risk of neonatal morbidity, including right ventricular failure, systemic hypotension, and multiorgan dysfunction [1]. Long-term consequences of PPHN can include neurodevelopmental impairments and chronic lung disease due to prolonged hypoxemia and the aggressive therapies often required. However, diagnosing PPHN can be challenging, as its symptoms overlap with other causes of neonatal respiratory distress, such as congenital heart disease and severe respiratory distress syndrome [4]. Moreover, the management of PPHN requires a multidisciplinary approach, with the timely use of specialized therapies like inhaled nitric oxide, extracorporeal membrane oxygenation (ECMO), and targeted pharmacological interventions, which may not be readily available in all healthcare settings [4].

This review aims to provide a comprehensive overview of the current pharmaceutical strategies used in the management of PPHN. This review will explore the various pharmacological agents available to reduce pulmonary vascular resistance, improve oxygenation, and stabilize the cardiovascular status of affected neonates. It will also examine the latest evidence supporting the use of these therapies, focusing on their mechanisms of action, clinical efficacy, safety profiles, and limitations. As our understanding of PPHN’s pathophysiology continues to evolve, so does the landscape of therapeutic options. This review aims to offer an up-to-date synthesis of the available literature, informing clinicians, researchers, and policymakers of the most effective and evidence-based pharmaceutical strategies for managing PPHN, aiming to improve outcomes for this vulnerable population.

## Review

Pathophysiology of PPHN

During the fetal stage, the placenta is the primary site for gas exchange, allowing the fetus to receive oxygen and expel carbon dioxide without relying on its lungs. Blood from the right ventricle is channeled through the ductus arteriosus into the descending aorta, bypassing the fluid-filled lungs [5]. At birth, the newborn undergoes a critical circulatory transition: pulmonary vascular resistance (PVR) decreases significantly as the lungs expand with air, while systemic vascular resistance increases. This transition allows most cardiac output to flow through the lungs, facilitating effective gas exchange and oxygenation [5]. In PPHN, the expected decrease in PVR after birth does not occur, resulting in persistently high resistance in the pulmonary arteries. This condition leads to inadequate blood flow to the lungs and severe hypoxemia due to right-to-left shunting of deoxygenated blood through the ductus arteriosus and foramen ovale. Several factors contribute to the increased PVR, including functional vasoconstriction and structural changes in the pulmonary vasculature [1]. Vasoconstriction is often mediated by factors such as endothelin-1 and thromboxane A2, which promote the narrowing of pulmonary arteries. Additionally, structural remodeling involves the proliferation of pulmonary artery smooth muscle cells and the deposition of extracellular matrix proteins, exacerbating the condition.

Endothelial dysfunction plays a central role in the pathogenesis of PPHN. Normally, the endothelium produces vasodilators like nitric oxide (NO) and prostacyclin to maintain low PVR [6]. In PPHN, however, these vasodilators have reduced production, and there is an increased production of vasoconstrictors such as endothelin-1. This imbalance results in persistent vasoconstriction and contributes to the structural remodeling of the pulmonary arteries, characterized by intimal thickening, medial hypertrophy, and adventitial fibrosis [6]. The molecular mechanisms underlying PPHN also involve the nitric oxide (NO) pathway, which is crucial for regulating pulmonary vascular tone. Reduced endothelial nitric oxide synthase (eNOS) expression and activity lead to decreased NO production, contributing to elevated PVR. Moreover, oxidative stress can further diminish NO bioavailability by promoting its inactivation, exacerbating pulmonary hypertension [2]. Genetic factors also play a significant role in the development of PPHN. Mutations in genes associated with the transforming growth factor-β (TGF-β) signaling pathway, particularly in the BMPR2 gene, have been linked to familial and idiopathic pulmonary arterial hypertension. Other genetic variants, such as those in SMAD9, NOTCH3, and CPS1, have also been associated with an increased risk of PPHN. Understanding these molecular and genetic factors is crucial for developing targeted therapies to improve outcomes for infants affected by this serious condition [7].

Diagnostic approaches

Diagnosing Persistent Pulmonary Hypertension in the Newborn (PPHN) requires a thorough approach encompassing clinical evaluation, imaging studies, biomarker analysis, and differential diagnosis considerations. Each component is essential for accurately identifying PPHN and distinguishing it from other conditions with similar presentations [8]. Clinically, PPHN can present with a range of symptoms, including cyanosis, respiratory distress, hypoxemia, heart murmurs, and decreased perfusion. Cyanosis, a bluish skin discoloration particularly noticeable in the lips and extremities, indicates inadequate oxygenation. Respiratory distress may include tachypnea (rapid breathing), grunting, nasal flaring, and retractions [1]. Hypoxemia, or low blood oxygen levels, often necessitates supplemental oxygen or mechanical ventilation. Heart murmurs may occur due to increased right-to-left shunting through the ductus arteriosus or foramen ovale. Decreased perfusion, as evidenced by cold extremities and poor capillary refill, may indicate compromised circulation, complicating the clinical picture [9].

Echocardiography is the primary imaging modality for diagnosing PPHN, providing critical information about the condition. It allows for assessing pulmonary artery pressure through Doppler studies, which estimate right ventricular systolic pressure and help gauge the severity of pulmonary hypertension. Echocardiography also evaluates cardiac function, assessing right and left ventricular performance and identifying any shunting through the foramen ovale or ductus arteriosus. Additionally, it can help rule out congenital heart defects that may mimic or contribute to PPHN [10]. While echocardiography is central to diagnosis, other imaging modalities may be used in specific scenarios. Magnetic resonance imaging (MRI) is less commonly used in acute settings but can provide detailed images of the heart and pulmonary vasculature, making it valuable for research contexts. Computed tomography (CT) scans are rarely employed in neonates due to radiation exposure but can be informative in selected cases, particularly for assessing anatomical abnormalities [11].

Research into biomarkers for PPHN is ongoing, with several potential candidates being explored. Notably, N-terminal pro-B-type Natriuretic Peptide (NT-proBNP) may be elevated in PPHN and correlate with the severity of pulmonary hypertension. Additionally, endothelin-1, a potent vasoconstrictor, might be increased in PPHN and could serve as a potential biomarker for the condition [12]. Arterial blood gas (ABG) analysis is crucial for evaluating the severity of hypoxemia and assessing acid-base status in affected infants. A low partial pressure of oxygen (PaO2) indicates inadequate oxygenation, while metabolic or respiratory acidosis can provide insights into the underlying physiological derangements and guide treatment decisions [13]. Differentiating PPHN from other causes of neonatal respiratory distress is vital for effective management. PPHN can resemble conditions such as respiratory distress syndrome (RDS), which is often due to surfactant deficiency in premature infants and is characterized by ground-glass opacities on chest X-ray [14]. Meconium Aspiration Syndrome (MAS) can also lead to airway obstruction and pulmonary inflammation, typically showing hyperinflation and atelectasis on imaging. Congenital heart disease, including transposition of the great vessels or coarctation of the aorta, can present with similar symptoms and requires echocardiographic evaluation for differentiation [14]. Pulmonary infections, such as pneumonia, can cause respiratory distress and may be accompanied by fever and abnormal lung sounds, necessitating careful consideration during diagnosis [14]. Diagnostic approaches for Persistent Pulmonary Hypertension of the Newborn (PPHN) are summarized in Table 1.

**Table 1:** Diagnostic approaches for PPHN PPHN: Persistent pulmonary hypertension of the newborn

Diagnostic Approach	Description	Advantages	Limitations
Clinical Presentation [15]	Observing signs and symptoms such as cyanosis, respiratory distress, and hypoxemia.	Non-invasive and quick to identify	Non-specific, overlaps with other neonatal conditions
Echocardiography [16]	Non-invasive imaging to assess pulmonary pressures and right-to-left shunting through the heart.	The gold standard for diagnosing PPHN	Operator dependent may not reveal all structural issues
Other Imaging Techniques [17]	MRI and CT can provide detailed structural information, particularly in complex cases.	High resolution for anatomical details	Requires transportation, high cost, not always feasible in unstable neonates
Biomarkers [18]	Blood tests to assess oxygenation and potential endothelial dysfunction (e.g., BNP, troponin levels).	Can support clinical diagnosis and assess the severity	Limited availability, research on specific markers still ongoing
Blood Gas Analysis [19]	Measures oxygenation and acid-base status to detect hypoxemia and acidosis.	Widely available and inexpensive	Cannot differentiate PPHN from other causes of hypoxemia
Differential Diagnosis [15]	Differentiating PPHN from other neonatal respiratory conditions like RDS and congenital heart disease.	Helps rule out other critical conditions	Requires a combination of tests for accurate diagnosis

Pharmaceutical interventions

Managing PPHN aims to achieve several critical therapeutic goals. The primary objective is to reduce pulmonary vascular resistance (PVR), which is essential for improving pulmonary blood flow and ensuring adequate oxygenation [1]. Healthcare providers strive to enhance cardiac output by lowering PVR, supporting the infant's overall hemodynamic stability. Additionally, preventing complications associated with PPHN, such as right ventricular failure and end-organ damage, is crucial for improving long-term outcomes in affected neonates. These therapeutic goals guide the selection and application of various pharmaceutical interventions in clinical practice [1]. Inhaled nitric oxide (iNO) is a cornerstone of PPHN management due to its potent pulmonary vasodilatory effects. It stimulates guanylate cyclase in vascular smooth muscle cells, increasing cyclic guanosine monophosphate (cGMP) levels and subsequent vasodilation [20]. Clinical evidence supports the use of iNO, with guidelines indicating its effectiveness in improving oxygenation and reducing the need for extracorporeal membrane oxygenation (ECMO) in term or near-term infants with PPHN. However, a Cochrane review in 2017 highlighted that while iNO significantly enhances oxygenation, it does not improve overall survival rates, and approximately 40% of neonates may not respond to the treatment [21].

Potential limitations and side effects of iNO therapy include methemoglobinemia and the risk of rebound pulmonary hypertension upon discontinuation, necessitating careful monitoring during treatment [21]. Phosphodiesterase inhibitors, particularly sildenafil, have emerged as valuable adjuncts in managing PPHN. Sildenafil inhibits phosphodiesterase type 5 (PDE5), leading to increased cGMP levels and subsequent vasodilation [22]. Clinical trials have shown that sildenafil can improve oxygenation and reduce the need for ECMO in neonates with PPHN, making it a promising therapeutic option. Additionally, combination therapies involving sildenafil and iNO may offer additive effects, particularly in cases where infants do not respond adequately to iNO alone. This approach underscores the importance of personalized treatment strategies to optimize outcomes for neonates with PPHN [22]. Endothelin receptor antagonists, such as bosentan, are another class of pharmacological agents being explored for PPHN management. Bosentan works by blocking endothelin receptors, leading to vasodilation and reduced PVR. Although the potential benefits of bosentan are recognized, evidence from neonatal studies is limited, primarily consisting of case reports and small clinical trials [23]. Further research is needed to establish the efficacy and safety of bosentan in the neonatal population with PPHN. The safety profile of bosentan must be carefully considered, as it may have side effects that could impact vulnerable neonates [23]. An overview of pharmaceutical interventions for PPHN is summarized in Table 2.

**Table 2:** Overview of pharmaceutical interventions for PPHN PPHN: Persistent pulmonary hypertension of the newborn

Pharmaceutical Intervention	Mechanism of Action	Clinical Evidence	Limitations / Side Effects
Inhaled Nitric Oxide (iNO) [24]	Stimulates guanylate cyclase in vascular smooth muscle cells, increasing cyclic guanosine monophosphate (cGMP) levels, leading to pulmonary vasodilation.	Proven to improve oxygenation and reduce the need for ECMO in term and near-term infants. Widely accepted as a first-line treatment.	Approximately 30-40% of infants may not respond. Possible side effects include methemoglobinemia and rebound pulmonary hypertension upon discontinuation.
Sildenafil (Phosphodiesterase Type 5 Inhibitor) [25]	Inhibits phosphodiesterase type 5 (PDE-5), increasing cGMP, leading to enhanced vasodilation in pulmonary vessels.	Shown to improve oxygenation and decrease ECMO need in PPHN. Used as an adjunct or in cases unresponsive to iNO. Ongoing clinical trials are evaluating long-term efficacy.	Risk of systemic hypotension, and the long-term safety in neonates remains unclear. Gastrointestinal disturbances have been reported.
Bosentan (Endothelin Receptor Antagonist) [26]	Blocks endothelin receptors (ETA and ETB), reducing vasoconstriction and decreasing pulmonary vascular resistance.	Limited neonatal studies; some case reports show benefit in severe PPHN cases unresponsive to standard therapies.	Risk of liver toxicity and drug interactions. Neonatal-specific data are still limited, and further research is required to validate safety and efficacy.
Prostacyclin Analogues (e.g., Epoprostenol, Treprostinil) [27]	Induces vasodilation by stimulating prostacyclin receptors, inhibiting platelet aggregation, and promoting pulmonary vascular relaxation.	Can improve oxygenation and reduce pulmonary artery pressure in selected cases. Often used when iNO is not available or ineffective.	Requires continuous infusion, which poses challenges in neonates. Side effects include hypotension, bleeding, and infection risks from catheter use.
Soluble Guanylate Cyclase (sGC) Stimulators (e.g., Riociguat) [28]	Enhances the nitric oxide-sGC-cGMP pathway, improving vasodilation and reducing pulmonary arterial pressure.	Early-stage clinical trials suggest promising outcomes in reducing pulmonary hypertension. Still under investigation for neonatal use.	Still experimental in neonates with limited safety and efficacy data. Potential for systemic hypotension and other side effects.
Rho-kinase Inhibitors [29]	Inhibits the Rho-kinase pathway, preventing vasoconstriction and reducing pulmonary vascular remodeling.	Preclinical studies indicate potential for decreasing pulmonary vascular resistance. Some promising results in early animal models.	Not yet widely available or clinically approved for neonatal use. More studies are required to assess safety in infants.
Corticosteroids [30]	Reduces inflammation and oxidative stress that contribute to pulmonary vascular remodeling and hypoxemia.	Limited neonatal data. May be used as adjunct therapy in specific cases of inflammation-related PPHN.	Risk of immunosuppression, impaired growth, and neurodevelopmental issues, especially with prolonged use.
Milrinone (Phosphodiesterase Type 3 Inhibitor) [30]	Increases intracellular cAMP, leading to vasodilation and improved cardiac function.	Some studies show beneficial effects in reducing pulmonary hypertension and improving cardiac output in neonates with PPHN.	Potential for systemic hypotension and arrhythmias. Still under investigation for routine use in PPHN.

Non-pharmaceutical interventions

Managing PPHN requires a comprehensive approach, incorporating both supportive care and advanced interventions such as extracorporeal membrane oxygenation (ECMO) [31]. Supportive care is pivotal in managing PPHN and involves several critical components. Oxygen therapy ensures adequate oxygen saturation levels, with continuous monitoring to prevent hypoxemia. Ventilation strategies, including mechanical and high-frequency ventilation (HFV), help maintain normal functional residual capacity and recruit collapsed lung areas [32]. It is important to avoid overexpansion of the lungs, as this can worsen pulmonary hypertension. For newborns with parenchymal lung disease, surfactant therapy is indicated. This treatment reduces surface tension in the alveoli and improves lung function, which is especially beneficial for premature infants or those with respiratory distress syndrome [1]. ECMO is a vital intervention for severe cases of PPHN when conventional therapies prove insufficient. It is recommended for infants with severe PPHN who do not respond to medical treatments like inhaled nitric oxide or other pulmonary vasodilators. Early initiation of ECMO is crucial to prevent irreversible damage and improve outcomes. ECMO can be combined with pharmacological treatments, such as inhaled nitric oxide and sildenafil, to enhance pulmonary vasodilation while providing essential respiratory support [33]. This integrated approach helps stabilize the infant's condition and allows time for lung recovery. ECMO has been associated with improved survival rates in severe PPHN cases. However, the long-term prognosis can vary; while many infants recover, a significant proportion may face long-term complications, including neurodevelopmental impairments and hearing difficulties. Ongoing follow-up is necessary to monitor developmental progress and address emerging issues [33]. An overview of non-pharmaceutical interventions for managing PPHN is detailed in Table 3.

**Table 3:** Non-pharmaceutical interventions for managing PPHN PPHN: Persistent pulmonary hypertension of the newborn

Non-Pharmaceutical Intervention	Description	Clinical Benefits	Limitations/Challenges
Oxygen Therapy [34]	Administering supplemental oxygen to improve oxygenation and reduce pulmonary vascular resistance.	Effective in reducing hypoxemia and improving pulmonary vasodilation.	Risk of oxygen toxicity, especially in preterm infants.
Ventilation Strategies (e.g., HFOV) [35]	High-frequency oscillatory ventilation (HFOV) improves gas exchange by minimizing lung injury.	Effective in managing severe hypoxemia when conventional methods fail.	Requires specialized equipment and expertise.
Surfactant Therapy [36]	Administration of surfactant to improve lung compliance and oxygenation in infants with lung disease.	Helps reduce alveolar collapse and improves oxygenation.	Limited to infants with associated respiratory distress syndrome (RDS).
Extracorporeal Membrane Oxygenation (ECMO) [37]	Mechanical support that oxygenates blood outside the body, bypassing the lungs.	Life-saving in severe PPHN when other treatments fail.	Invasive, high risk of complications (e.g., bleeding, infection), high cost.
Supportive Care (e.g., Fluid Management) [38]	Ensuring appropriate hydration, electrolyte balance, and cardiovascular support to maintain stability.	Provides a stable environment for effective medical treatment.	Requires careful monitoring to prevent fluid overload or dehydration.
Sedation and Pain Management [39]	Reducing stress and agitation through appropriate use of sedation to decrease oxygen consumption.	Improves oxygenation by minimizing stress and metabolic demand.	Risk of prolonged sedation leading to respiratory depression.

Challenges in current management

Persistent Pulmonary Hypertension of the Newborn (PPHN) is a severe condition marked by elevated pulmonary vascular resistance, impairs blood flow to the lungs and leads to hypoxemia. PPHN can arise from complications such as congenital diaphragmatic hernia or meconium aspiration syndrome, or it may occur idiopathically. Management strategies for PPHN have advanced significantly, focusing on optimizing pulmonary blood flow and minimizing hypoxic damage [40]. A key component of PPHN management is using pulmonary vasodilators, which help reduce pulmonary vascular resistance and improve oxygenation [20]. Principal agents include inhaled nitric oxide (iNO), the most commonly used pulmonary vasodilator for PPHN; sildenafil, an oral and intravenous phosphodiesterase type 5 inhibitor; and prostaglandin E1, which is occasionally used to maintain ductal patency in infants with concurrent ductus arteriosus-dependent circulation. Supportive care is also critical, encompassing strategies to optimize oxygenation, prevent acidosis, and, in severe cases, use extracorporeal membrane oxygenation (ECMO) [20]. Despite advancements, several challenges in managing PPHN persist and can impact patient outcomes. Treatment response can vary widely among neonates due to genetic factors and environmental influences [1].

Genetic variations may affect both the pulmonary vasculature and the metabolism of medications. At the same time, factors such as maternal health, prenatal exposure to drugs or infections, and the infant's postnatal environment can influence PPHN severity and treatment response. Furthermore, PPHN severity can differ among patients, depending on underlying conditions and the timing of diagnosis [1]. Pharmacological treatments for PPHN, while effective, are not without risks. Inhaled nitric oxide, for instance, can lead to methemoglobinemia, and sildenafil may cause systemic hypotension or gastrointestinal disturbances. Continuous monitoring is essential to identify and manage these side effects promptly. The need for ongoing adjustment of treatment regimens can complicate care in the neonatal intensive care unit (NICU), necessitating a multidisciplinary approach [41]. Long-term outcomes for infants with PPHN are significant and warrant careful attention. These infants are at increased risk for neurodevelopmental delays and disabilities, with factors such as the severity of hypoxemia, duration of mechanical ventilation, and use of ECMO playing a role in these outcomes [1]. Long-term follow-up is crucial for monitoring developmental progress and providing early interventions. However, predicting which infants are at higher risk for long-term complications remains challenging due to outcome variability [1]. An overview of the challenges in the current management of PPHN is detailed in Table 4.

**Table 4:** Challenges in the current management of PPHN PPHN: Persistent pulmonary hypertension of the newborn

Challenge	Description	Impact on Management	Potential Solutions
Variability in Treatment Response [42]	Differences in how neonates respond to therapies due to genetic, environmental, and disease-specific factors.	Makes it difficult to predict outcomes and optimize treatments for all patients.	Personalized medicine approaches use genetic and biomarker data to guide therapy.
Adverse Effects of Therapies [43]	Side effects of medications like nitric oxide, sildenafil, and ECMO (e.g., hypotension, bleeding, neurodevelopmental effects).	Long-term side effects can impact overall prognosis and quality of life.	Improved monitoring of side effects and development of safer drugs with fewer long-term effects.
Variability in Disease Severity [1]	PPHN ranges from mild to life-threatening, making a uniform treatment protocol challenging.	It requires tailored interventions based on severity, which complicates treatment plans.	Stratified care approaches that categorize patients based on risk and severity.
Delayed Diagnosis [1]	Early signs of PPHN can be subtle, leading to delays in diagnosis and intervention.	Delayed treatment increases the risk of poor outcomes and complications.	Enhanced screening protocols and training for early identification of PPHN.
Limited Long-Term Outcome Data [44]	Few studies focus on long-term neurodevelopmental and physical outcomes post-PPHN treatment.	Lack of data hinders understanding of prognosis and potential developmental delays.	Longitudinal studies track survivors of PPHN to assess long-term impacts.
High Cost of Advanced Therapies [45]	Treatments like ECMO and nitric oxide are expensive and require specialized equipment.	Limits availability, especially in low-resource settings, affecting access to life-saving treatments.	Cost-reduction strategies, development of alternative therapies, and improving access to affordable care.

Future directions and research

Researchers are actively investigating new therapeutic agents that target alternative pathways in the pathogenesis of PPHN. Among these, L-citrulline, an amino acid, has demonstrated potential in experimental models by enhancing nitric oxide production and improving pulmonary vasodilation [46]. Endothelin receptor antagonists, such as bosentan, which block endothelin-1-mediated vasoconstriction, are also under investigation, though clinical trial results have been mixed. Additionally, soluble guanylyl cyclase (sGC) stimulators and activators, which enhance the nitric oxide signaling pathway, have shown efficacy in animal studies. Rho kinase inhibitors, which disrupt the regulation of vascular smooth muscle contraction, represent a novel approach to treating PPHN [46]. Given the diverse etiologies of PPHN, personalized treatment strategies targeting specific pathways may prove more effective than a universal approach. Current research is focused on identifying predictive biomarkers and utilizing pharmacogenomics to tailor therapies [2]. Identifying biomarkers that predict responses to treatments like inhaled nitric oxide or sildenafil could enable clinicians to personalize care and avoid ineffective therapies. Potential biomarkers include indicators of oxidative stress, inflammation, and endothelial dysfunction. Pharmacogenomic studies aim to uncover genetic variants that affect drug metabolism and response in PPHN, potentially leading to genotype-guided dosing that optimizes efficacy while minimizing toxicity [47].

In addition to traditional pharmacological interventions, emerging research is exploring the potential of gene therapy and stem cell-based treatments for PPHN. Gene therapy approaches aim to correct or modulate the underlying genetic abnormalities contributing to pulmonary hypertension. For instance, experimental models have investigated the use of gene transfer to enhance the expression of endothelial nitric oxide synthase (eNOS) or other vasodilatory agents in the pulmonary vasculature. While still in the early stages, these strategies hold promise for providing long-lasting therapeutic effects by directly addressing the molecular defects in PPHN. Stem cell therapy, particularly with mesenchymal stem cells (MSCs), is another area of interest. MSCs have shown the potential to promote vascular repair, modulate inflammation, and improve lung function in preclinical models of neonatal lung injury. Ongoing studies are exploring whether MSCs can be harnessed to mitigate pulmonary vascular remodeling and promote healing in PPHN.

Although these therapies are still experimental and face significant challenges related to safety, delivery, and efficacy, they represent a frontier for future therapeutic interventions. Several clinical trials are underway to assess novel therapies for PPHN. For instance, the SHIP trial is evaluating the efficacy and safety of intravenous sildenafil in infants with PPHN. The FUTURE-4 trial investigates the use of bosentan in infants with PPHN who are resistant to inhaled nitric oxide. Preclinical studies suggest that L-citrulline may enhance nitric oxide production, with clinical trials planned to explore this potential [48]. As new therapies emerge and evidence evolves, updating international guidelines for PPHN management will be essential. Such updates will help standardize care and inform clinicians of the latest treatment options. Ongoing research focuses on developing innovative pharmacological agents, integrating biomarkers and genomics to personalize therapy, and exploring advanced therapeutic modalities such as gene therapy and stem cell treatments. Conducting well-designed clinical trials and updating international guidelines will be crucial for translating research findings into clinical practice and improving outcomes for infants with this challenging condition [33]. Future directions and research in managing PPHN are detailed in Table 5.

**Table 5:** Future directions and research in the management of PPHN PPHN: Persistent pulmonary hypertension of the newborn

Future Direction	Description	Potential Impact	Current Limitations
Innovations in Drug Development [49]	Development of novel therapies targeting specific molecular pathways, such as Rho-kinase inhibitors and soluble guanylate cyclase stimulators.	It may offer more targeted treatments with fewer side effects and greater efficacy in neonates.	Limited clinical trials and safety data for neonates.
Personalized Medicine Approaches [1]	Tailoring treatments based on genetic and molecular profiling of neonates with PPHN to improve efficacy.	Potential to optimize outcomes by delivering individualized therapies based on specific patient characteristics.	High cost and need for advanced genetic testing.
Integration of Biomarkers [50]	Use predictive biomarkers (e.g., BNP, troponin) to guide early diagnosis, assess disease severity, and predict treatment response.	It could enable early intervention and more precise treatment monitoring, improving outcomes.	Lack of standardized biomarkers for widespread clinical use.
Genomic Research and Therapy [51]	Exploring the role of genetic predispositions and gene therapy as a potential avenue for treating PPHN.	It can help identify at-risk neonates and develop preventive or curative treatments based on genetic insights.	Research is still in the early stages; ethical considerations.
Clinical Trials and Updated Guidelines [52]	Ongoing and future clinical trials aimed at refining current treatment protocols and introducing novel therapies into standard care.	New trials can improve treatment efficacy, provide evidence for new guidelines, and refine best practices.	Long approval process for new drugs and therapies, with variability in trial outcomes.
Advances in Non-Invasive Monitoring [53]	Development of advanced imaging and non-invasive monitoring techniques to assess pulmonary pressures and oxygenation more accurately.	It could reduce the need for invasive procedures and provide continuous monitoring, improving patient safety.	High cost of equipment and lack of widespread availability in all healthcare settings.

## Conclusions

In conclusion, PPHN remains a formidable challenge in neonatal care, with significant implications for both short- and long-term outcomes. The condition's complexity arises from its multifactorial pathophysiology, involving disrupted pulmonary vascular transition, endothelial dysfunction, and a host of genetic and environmental factors. Early and accurate diagnosis is critical, yet the overlap of PPHN symptoms with other neonatal conditions often complicates it. Current pharmaceutical strategies, including inhaled nitric oxide, prostacyclin analogs, phosphodiesterase inhibitors, and advanced therapies like ECMO, have significantly improved survival rates. However, these treatments are not without limitations, and their efficacy can vary depending on the individual patient's condition and the timing of intervention.

As new therapies and drug delivery systems emerge, ongoing research and clinical trials are essential to refine these strategies and develop more targeted, effective treatments. From a global perspective, the management of PPHN presents unique challenges, particularly in low-resource settings where access to advanced therapies such as inhaled nitric oxide and ECMO is often limited. In these regions, the high cost of medications, lack of specialized equipment, and inadequate healthcare infrastructure can significantly impede the effective management of PPHN, contributing to higher morbidity and mortality rates. Strategies to improve care worldwide include enhancing the availability and affordability of essential medications, developing cost-effective alternatives to advanced therapies, and implementing training programs to improve the early diagnosis and management of PPHN in resource-limited settings. Collaborations between global health organizations, governments, and local healthcare providers are crucial to addressing these disparities and ensuring that all newborns, regardless of their geographical location, have access to life-saving treatments. Ultimately, a multidisciplinary approach integrating the latest pharmaceutical advances with early diagnosis, comprehensive care, and global health initiatives is key to improving outcomes for newborns affected by PPHN.

## References

[1] Nandula PS, Shah SD (2024). Persistent pulmonary hypertension of the newborn. https://www.ncbi.nlm.nih.gov/books/NBK585100/.

[2] Martinho S, Adão R, Leite-Moreira AF, Brás-Silva C (2020). Persistent pulmonary hypertension of the newborn: pathophysiological mechanisms and novel therapeutic approaches. Front Pediatr.

[3] Steinhorn RH (2010). Neonatal pulmonary hypertension. Pediatr Crit Care Med.

[4] Lakshminrusimha S, Keszler M (2015). Persistent pulmonary hypertension of the newborn. Neoreviews.

[5] Remien K, Majmundar SH (2024). Physiology, fetal circulation. https://www.ncbi.nlm.nih.gov/books/NBK539710/.

[6] Makino A, Firth AL, Yuan JX (2011). Endothelial and smooth muscle cell ion channels in pulmonary vasoconstriction and vascular remodeling. Compr Physiol.

[7] Dai L, Du L (2022). Genes in pediatric pulmonary arterial hypertension and the most promising BMPR2 gene therapy. Front Genet.

[8] Sharma V, Berkelhamer S, Lakshminrusimha S (2015). Persistent pulmonary hypertension of the newborn. Matern Health Neonatol Perinatol.

[9] Gillam-Krakauer M, Mahajan K (2024). Patent ductus arteriosus. https://www.ncbi.nlm.nih.gov/books/NBK430758/.

[10] Augustine DX, Coates-Bradshaw LD, Willis J (2018). Echocardiographic assessment of pulmonary hypertension: a guideline protocol from the British Society of Echocardiography. Echo Res Pract.

[11] Shah S, Chryssos ED, Parker H (2009). Magnetic resonance imaging: a wealth of cardiovascular information. Ochsner J.

[12] Rodolaki K, Pergialiotis V, Sapantzoglou I (2023). N-terminal pro-b type natriuretic peptide as a predictive biomarker of bronchopulmonary dysplasia or death due to bronchopulmonary dysplasia in preterm neonates: a systematic review and meta-analysis. J Pers Med.

[13] Sood P, Paul G, Puri S (2010). Interpretation of arterial blood gas. Indian J Crit Care Med.

[14] Reuter S, Moser C, Baack M (2014). Respiratory distress in the newborn. Pediatr Rev.

[15] Yadav S, Lee B, Kamity R (2024). Neonatal respiratory distress syndrome. https://www.ncbi.nlm.nih.gov/books/NBK560779/.

[16] Topyła-Putowska W, Tomaszewski M, Wysokiński A, Tomaszewski A (2021). Echocardiography in pulmonary arterial hypertension: comprehensive evaluation and technical considerations. J Clin Med.

[17] Hussain S, Mubeen I, Ullah N (2022). Modern diagnostic imaging technique applications and risk factors in the medical field: a review. Biomed Res Int.

[18] Chan D, Ng LL (2010). Biomarkers in acute myocardial infarction. BMC Med.

[19] Williams AJ (1998). Assessing and interpreting arterial blood gases and acid-base balance. BMJ.

[20] Singh Y, Lakshminrusimha S (2021). Pathophysiology and management of persistent pulmonary hypertension of the newborn. Clin Perinatol.

[21] Sokol GM, Konduri GG, Van Meurs KP (2016). Inhaled nitric oxide therapy for pulmonary disorders of the term and preterm infant. Semin Perinatol.

[22] Padda IS, Tripp J (2024). Phosphodiesterase inhibitors. https://www.ncbi.nlm.nih.gov/books/NBK559276/.

[23] More K, Athalye-Jape GK, Rao SC, Patole SK (2016). Endothelin receptor antagonists for persistent pulmonary hypertension in term and late preterm infants. Cochrane Database Syst Rev.

[24] Witek J, Lakhkar AD (2024). Nitric oxide. https://www.ncbi.nlm.nih.gov/books/NBK554485/.

[25] Liu C, Chen J, Gao Y, Deng B, Liu K (2013). Endothelin receptor antagonists for pulmonary arterial hypertension. Cochrane Database Syst Rev.

[26] Barnes H, Yeoh HL, Fothergill T, Burns A, Humbert M, Williams T (2019). Prostacyclin for pulmonary arterial hypertension. Cochrane Database Syst Rev.

[27] Lian TY, Jiang X, Jing ZC (2017). Riociguat: a soluble guanylate cyclase stimulator for the treatment of pulmonary hypertension. Drug Des Devel Ther.

[28] Duong-Quy S, Bei Y, Liu Z, Dinh-Xuan AT (2013). Role of Rho-kinase and its inhibitors in pulmonary hypertension. Pharmacol Ther.

[29] Yasir M, Goyal A, Sonthalia S (2024). Corticosteroid adverse effects. https://www.ncbi.nlm.nih.gov/books/NBK531462/.

[30] Makdisi G, Wang IW (2015). Extra corporeal membrane oxygenation (ecmo) review of a lifesaving technology. J Thorac Dis.

[31] Bhagwat AP, Sharath HV, Seth NH, Puri SN (2024). Persistent pulmonary hypertension of newborns secondary to labile hypoxemia associated with cyanosis: a case series. Cureus.

[32] Sharma M, Callan E, Konduri GG (1984). Pulmonary vasodilator therapy in persistent pulmonary hypertension of the newborn (PPHN). Clin Perinatol.

[33] Nair J, Lakshminrusimha S (2014). Update on PPHN: mechanisms and treatment. Semin Perinatol.

[34] Kayton A, Timoney P, Vargo L, Perez JA (2018). A review of oxygen physiology and appropriate management of oxygen levels in premature neonates. Adv Neonatal Care.

[35] Murthy PR, Kumar AK A (2024). High frequency ventilation. https://www.ncbi.nlm.nih.gov/books/NBK563151/.

[36] Khawar H, Marwaha K (2024). Surfactant. https://www.ncbi.nlm.nih.gov/books/NBK546600/.

[37] Vyas A, Bishop MA (2024). Extracorporeal membrane oxygenation in adults. https://www.ncbi.nlm.nih.gov/books/NBK576426/.

[38] Malbrain ML, Langer T, Annane D (2020). Intravenous fluid therapy in the perioperative and critical care setting: executive summary of the International Fluid Academy (IFA). Ann Intensive Care.

[39] Oddo M, Crippa IA, Mehta S, Menon D, Payen JF, Taccone FS, Citerio G (2016). Optimizing sedation in patients with acute brain injury. Crit Care.

[40] Mathew B, Lakshminrusimha S (2017). Persistent pulmonary hypertension in the newborn. Children (Basel).

[41] Dhariwal AK, Bavdekar SB (2015). Sildenafil in pediatric pulmonary arterial hypertension. J Postgrad Med.

[42] Singh AV, Chandrasekar V, Paudel N (2023). Integrative toxicogenomics: Advancing precision medicine and toxicology through artificial intelligence and OMICs technology. Biomed Pharmacother.

[43] Smith BP, Babos M (2024). Sildenafil. https://www.ncbi.nlm.nih.gov/books/NBK558978/.

[44] Rohana J, Boo NY, Chandran V, Sarvananthan R (2011). Neurodevelopmental outcome of newborns with persistent pulmonary hypertension. Malays J Med Sci.

[45] Oude Lansink-Hartgring A, van Minnen O, Vermeulen KM, van den Bergh WM (2021). Hospital costs of extracorporeal membrane oxygenation in adults: a systematic review. Pharmacoecon Open.

[46] Christou H, Khalil RA (2022). Mechanisms of pulmonary vascular dysfunction in pulmonary hypertension and implications for novel therapies. Am J Physiol Heart Circ Physiol.

[47] Cipak Gasparovic A, Zarkovic N, Zarkovic K, Semen K, Kaminskyy D, Yelisyeyeva O, Bottari SP (2017). Biomarkers of oxidative and nitro-oxidative stress: conventional and novel approaches. Br J Pharmacol.

[48] Pierce CM, Zhang MH, Jonsson B, Iorga D, Cheruvu N, Balagtas CC, Steinhorn RH (2021). Efficacy and safety of IV sildenafil in the treatment of newborn infants with, or at risk of, persistent pulmonary hypertension of the newborn (PPHN): a multicenter, randomized, placebo-controlled trial. J Pediatr.

[49] Shah AJ, Beckmann T, Vorla M, Kalra DK (2023). New drugs and therapies in pulmonary arterial hypertension. Int J Mol Sci.

[50] Méndez Hernández R, Ramasco Rueda F (2023). Biomarkers as prognostic predictors and therapeutic guide in critically ill patients: clinical evidence. J Pers Med.

[51] Liu X, Mei M, Chen X (2019). Identification of genetic factors underlying persistent pulmonary hypertension of newborns in a cohort of Chinese neonates. Respir Res.

[52] Li A, Bergan RC (2020). Clinical trial design: past, present, and future in the context of big data and precision medicine. Cancer.

[53] van Wijk JJ, Weber F, Stolker RJ, Staals LM (2020). Current state of noninvasive, continuous monitoring modalities in pediatric anesthesiology. Curr Opin Anaesthesiol.

